# Expression of nerve growth factor in the prostate of male rats in response to chronic stress and sympathetic denervation

**DOI:** 10.3892/etm.2014.1856

**Published:** 2014-07-21

**Authors:** SHENGLIANG HUANG, XUEBEI ZHANG, LIUYU XU, QING LI, QINGLI ZHAO

**Affiliations:** 1 Department of Urology, Shandong Provincial Qianfoshan Hospital, Shandong University, Jinan, Shandong 250014, P.R. China; 2 Department of Emergency, Qilu Hospital of Shandong University, Jinan, Shandong 250012, P.R. China

**Keywords:** chronic stress, nerve growth factor, prostatic disease, sympathetic denervation, Wistar rat

## Abstract

Nerve growth factor (NGF) has been found in the normal prostate of the Wistar rat and is regarded as an important prostatic mitogen. We have previously shown that chronic stress induced epithelial hyperplasia while sympathetic denervation caused atrophy in the male Wistar rat prostate. NGF may have been a contributing mechanism to the hyperplasia and atrophy response that was observed. The aim of the present study was to investigate the expression of NGF in the prostate of the male rat in response to chronic stress and denervation. Two weeks of restraint water-immersion stress were used to induce a chronic stress model in Wistar rats. Denervation of the peripheral sympathetic nerve was induced by 6-hydroxydopamine. The expression levels of NGF in the dissected prostate lobes were examined by immunohistochemistry. After 14 days of stress, proliferation of the epithelium in the ventral lobes was observed, whereas the dorsolateral lobes were almost unaffected. NGF immunoreactive protein was localized to the columnar secretory epithelium lines of the prostate tissue. Stress and denervation led to an increase in NGF expression in the ventral lobes. In conclusion, NGF was involved in the hyperplasia and atrophy in the prostate of the male rat in response to chronic stress and sympathetic denervation, and thus may be a contributing factor in the pathophysiology of the prostate.

## Introduction

Nerve growth factor (NGF) has essential roles in the survival, development and differentiation of neurons in the nervous system (1). It is known that NGF also plays an important role in a variety of non-neuronal systems. NGF and its receptors have been identified in normal prostatic tissue, benign prostatic hyperplasia and prostatic cancer, suggesting a potential role in the cell biology of these tissues (2).

The prostate is the second most abundant source of NGF following the central nervous system (3). In order for NGF to have an antiproliferative effect, it is crucial that two NGF receptors, namely p75 and tropomyosin-related kinase A are coexpressed. In prostate tumorigenesis the expression of p75 is increasingly lost and its disappearance is an indication of prostate adenocarcinoma. Additionally, dysregulation of NGF signal transduction was identified in a number of human tumors (4). NGF may also contribute to benign proliferation, as it has been observed in chronic prostate and chronic pelvic pain syndrome, since the abundance of NGF in prostate secretions varied in proportion to pain severity.

It has been reported that NGF expression is absent or present at low levels in the ventral rat prostate, whereas basal levels of expression are present in the dorsal rat prostate. Denervation of the rat prostate induces the expression of NGF in the ventral prostate and increases the level of its expression in the dorsal prostate (5). It has previously been suggested that NGF localizes to the columnar secretory epithelium lines of prostate tissue in rats (6).

Our previous study showed that two weeks of stress induced by restraint water-immersion stress (WIRS) led to hyperplastic morphological abnormalities in the ventral prostate of adult male Wistar rats (7). In addition, chemical sympathectomy with 6-hydroxydopamine (6-OHDA) promoted the dilation of prostatic alveoli and atrophy of the epithelium in all prostatic lobes.

In the present study, we hypothesized that NGF was involved in the hyperplasia and atrophy responses observed in our previous study. The purpose of the present study was to investigate the changes in NGF levels in the prostate of the male rat in response to chronic stress and sympathetic denervation.

## Materials and methods

### Animals and ethics statement

This study was carried out in strict accordance with the recommendations in the Guide for the Care and Use of Laboratory Animals of Shandong University (Jinan, China). The protocol was approved by the Committee on the Ethics of Animal Experiments of Shandong University (permit number, ECAESDUSM 20123008). All surgery was performed under chloral hydrate anesthesia, and all efforts were made to minimize suffering.

Male Wistar rats were purchased from the Animal Center of Shandong University and were maintained on standard laboratory food and water *ad libitum* with a 12-h light/dark photoperiod. The rats were raised for one week and handled for 2 min daily to facilitate acclimation. The chemical sympathectomy model with 6-OHDA (Sigma-Aldrich, St. Louis, MO, USA), as described by Vaalasti *et al* (8), was used on the ventral prostate of the rat, and the rats were additionally subjected to WIRS, as described previously (9). Sexually mature rats (90 days of age) were divided into four groups of eight rats each, as follows: i) Untreated control group; ii) WIRS group (WIRS for 1 h/day for two weeks); iii) 6-OHDA group (6-OHDA administered intravenously on days 1 and 8 at a dose of 50 mg/kg); and iv) WIRS plus 6-OHDA group (WIRS daily plus 6-OHDA on days 1 and 8 as above). The experiment was terminated at 14 days; animals were anesthetized by chloral hydrate intraperitoneally and then sacrificed by exsanguination.

The prostatic lobes were exposed immediately by a mid-abdominal incision. The ventral, lateral and dorsal prostatic lobes of the rat were removed separately and immediately fixed in 10% formaldehyde for histology and immunohistochemical observation.

### Histopathology processing

The prostatic lobes were fixed in 10% formalin solution for 10 h at room temperature. Thereafter, the samples were routinely processed. Paraffin-embedded sections of 4 μm were cut and stained by Harris’ hematoxylin and eosin.

### Immunohistochemistry

The paraffin-embedded tissues were sectioned at 4 μm thickness and mounted on poly-L-lysine-coated glass slides for >24 h at 32°C. The sections were deparaffinized with xylene and rehydrated in graded ethanol, then exposed to a microwave oven at 100°C for 15 min in sodium citrate buffer (0.01 M, pH 6.0) for antigen retrieval. Antigen retrieval was followed by incubation in 3% H_2_O_2_ at room temperature and then in 5% bovine serum albumin confining liquid. The tissue sections were subsequently incubated overnight at 37°C with polyclonal antibodies (1:100) against NGF (Boster Biological Technology Co., Ltd., Wuhan, China). The negative control sections were incubated with phosphate-buffered saline (PBS) in the absence of the primary antibody for each reaction.

For the immunoreaction, biotinylated goat anti-rabbit secondary antibodies and streptavidin-biotin complex (Boster Biological Technology Co., Ltd.) were used and the color reaction was detected using 3,3′-diaminobenzidine-H_2_O_2_ substrate according to the manufacturer’s instructions. The semi-quantitative assessment of NGF expression was performed using the Image-Pro Plus software 6.0 (Media Cybernetics, Inc., Rockville, MD, USA). The positive area was expressed by mean optical density.

### Statistical analysis

The evaluation of differences among the groups was performed using SPSS 13.0 (SPSS, Inc., Chicago, IL, USA). The statistical analysis was conducted using the two-way analysis of variance test, and P≤0.05 was considered to indicate a statistically significant difference.

## Results

**Figure 1:**
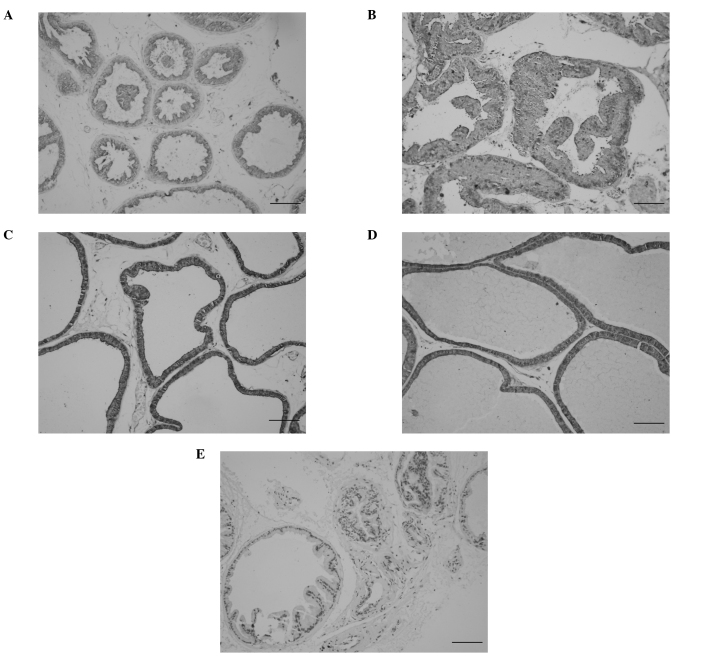
Expression of NGF in the prostatic lobes of male Wistar rats. (A) Ventral prostate of an untreated control rat. (B) Proliferation of the epithelium in the ventral prostate of a WIRS-treated rat, characterized by intraluminal villous enfolding, as well as the appearance of epithelial nodules, piling up of epithelial cells and a loss of cell polarity. NGF expression is increased. (C) Ventral prostate of a 6-OHDA-treated rat showing dilated acini and a significant increase in NGF level. (D) Ventral prostate of a WIRS plus 6-OHDA-treated rat without proliferation. (E) Negative control. All images were captured using the same magnification (original magnification, ×200; bar, 100 μm) and stained using Hematoxylin-Eosin. NGF, nerve growth factor; WIRS, restraint water-immersion stress; 6-OHDA, 6-hydroxydopamine.

In the untreated control rats, the acini of each prostatic lobe were rounded with secretion and lined by a single layer of epithelium. An immunopositive reaction for NGF was detected in the columnar secretory epithelium lines of the prostate (Fig. 1A). Chronic stress induced a proliferative change in the epithelium of the ventral lobes, which was characterized by intraluminal villous enfolding, as well as the appearance of epithelial nodules, piling up of epithelial cells and a loss of cell polarity (Fig. 1B). Chemical sympathectomy led to the dilation of the prostatic alveoli and atrophy of the epithelium in all lobes (Fig. 1C). The level of NGF increased significantly following stress or denervation in the ventral lobes, with higher levels following the latter (P<0.05). The dorsolateral lobes were almost unaffected in response to stress and denervation (Table I). In the WIRS plus 6-OHDA group, the ventral prostatic acini were dilated and no hyperplasia was observed. In this group, the NGF level in the ventral lobes was higher than that in the lobes in the untreated group (Fig. 1D). No immunostaining was detected in the control sections in which PBS was substituted for the primary antibody (Fig. 1E).

## Discussion

Several species, including humans, exhibit a relative abundance of NGF within the prostate (10,11). Prostate tissue derived from normal, benign prostatic hyperplasia and adenocarcinoma specimens has indicated that NGF immunoreactive protein is localized in the stromal compartment (12). NGF peptides play a physiological role in the control of prostate epithelial cell growth and differentiation in a paracrine interactive manner (11). A previous study showed that immunoreactive NGF was localized in the prostatic epithelium lines of adult male rats (6). This is consistent with the data in the present study.

Conflicting findings have been reported regarding the expression of NGF induced by denervation. The analysis of NGF expression in the rat prostate by quantitative polymerase chain reaction techniques showed that this neurotrophin was absent or present at low levels in the ventral prostate and present at basal levels in the dorsal prostate. Furthermore, denervation of the rat prostate induced the expression of NGF in the ventral prostate and increased the level of its expression in the dorsal prostate (5). Mesenteric arterial denervation significantly reduced the NGF levels in the adventitial layer and ganglia. Loss of the arterial nerve plexus resulted in a reduction in NGF expression levels; however, NGF content in the artery was not altered by sympathetic decentralization (13).

Our previous study demonstrated that sympathetic nervous system overactivity is involved in the pathogenesis of hyperplasia in the rat ventral prostate induced by chronic stress (7). Chemical sympathectomy caused apparent atrophy of the prostatic epithelium. NGF levels increased in the ventral prostate following chronic stress or denervation. The dorsal and lateral lobes were almost unaffected. These findings suggest that NGF may be a contributing mechanism to the hyperplasia in ventral lobes. Denervation leads to a significant increase in NGF expression levels in these lobes, which suggests that NGF has the physiological function of attempting to re-establish appropriate innervation. A previous study revealed that NGF could prevent prostate tumor growth through an indirect effect, possibly innervation of the tumor neovasculature (14).

Hyperplastic morphological abnormalities in the ventral prostate that progress with age have been observed in the spontaneously hypertensive rat (SHR), a genetic model of hypertension (15). SHRs exhibit excessive basal and environmentally evoked sympathetic activity and demonstrate increased production of NGF by smooth muscles such as the bladder (16). Kim *et al* (17) demonstrated the elevation of urinary NGF levels in patients with an overactive bladder. An increase in organ hyperinnervation is associated with NGF overproduction; this can affect gland growth either directly or in a neurally mediated manner.

It has been indicated that growth factor-mediated pathways are modified during the development and progression of prostate cancer (18). NGF has a mitogenic effect on the prostate. The autocrine expression of NGF has been revealed to be upregulated in the androgen refractory TSU-prl cell line, which is a human prostate epithelial tumor cell line (19).

In conclusion, the present study has demonstrated alterations in the expression of NGF in the adult rat prostate in response to stress and sympathetic denervation. Sympathetic nervous system overactivity resulted in hyperplasia of the ventral prostate and an increase in NGF expression, while denervation led to atrophy of the epithelium and higher NGF levels in the ventral lobes. NGF may thus be a contributing factor in the pathophysiology of the prostate.

## Figures and Tables

**Table I:** Expression of nerve growth factor in prostatic lobes measured in different groups.

Prostate region	Untreated (OD)	WIRS (OD)	6-OHDA (OD)	WIRS+6-OHDA (OD)
Ventral prostate	132.9±2.3	139.4±5.1^a^	146.2±6.2^a^,^b^	140.4±7.0^a^
Lateral prostate	135.0±3.3	133.0±7.0	137.0±5.6	138.1±3.5
Dorsal prostate	133.1±9.7	126.2±5.6	131.0±5.6	130.2±6.6

Data are presented as the mean ± standard error of the mean.

a P<0.05 vs. the untreated group;

b P<0.05 vs. the WIRS group.

WIRS, restraint water-immersion stress; 6-OHDA, 6-hydroxydopamine; OD, optical density.
